# Revalidation of Synonymy between *Nesotriatoma flavida* and *N. bruneri* (Hemiptera, Reduviidae, Triatominae)

**Published:** 2017-12-30

**Authors:** Oscar Páez-Rondón, Fernando Otálora-Luna, Elis Aldana

**Affiliations:** 1 Unidad de Articulación Comunitaria, Centro Multidisciplinario de Ciencias, Instituto Venezolano de Investigaciones Científicas, República Bolivariana de Venezuela; 2 Laboratorio de Ecología Sensorial, Centro Multidisciplinario de Ciencias, Instituto Venezolano de Investigaciones Científicas, República Bolivariana de Venezuela; 3 Laboratorio de Entomología “Herman Lent”, Departamento de Biología, Facultad de Ciencias, Universidad de Los Andes, República Bolivariana de Venezuela

**Keywords:** Chagas’ disease, Phallosome, Morphology, Taxonomy

## Abstract

**Background:**

We analyzed the external morphology and the external male genitalia of specimens of *Nesotriatoma flavida* of a laboratory colony founded with specimens from Guanahacabiles in Pinar del Río Province in the west of Cuba in 1980. This species was at first identified as different from *N. bruneri* and then later considered synonymous.

**Methods:**

We proposed to revise the morphological characters with which these species were considered as different and then later synonymous, such as the *fossula spongiosa* or spongy grooves, length of the first antenna segment, relationship length of eye to interocular distance, the form of the phallosome, phallosome support, and the endosome processes.

**Results:**

The results of the analyses of these characters in the specimens of our colony, and of the corresponding works where the separation and synonymy of these species has been proposed, allow us to sustain the revalidation of the synonymy between *N. flavida* and *N. bruneri*.

**Conclusion:**

Based on the body external morphology and the male external genitalia of *N. flavida* it is concluded that *N. flavida* and *N. bruneri* are synonymous species.

## Introduction

A new genus was describes, *Nesotriatoma*, in which he includes *Nesotriatoma flavida* (initially described as *Triatoma flavida*) collected in the western region of the island of Cuba and also a new species *Nesotriatoma bruneri*, collected in the eastern region of this island (1). *Nesotriatoma flavida* has a short first segment of the antenna that does not reach the peak of the clypeus, well developed spongy grooves on the front legs of the females, and eyes as wide as a third of the interocular distance. *Nesotriatoma bruneri* describes as having a long first antenna segment, reaching or passing the peak of the clypeus, no spongy grooves on the front legs of the females, and the width of the eyes as more than half the interocular distance. However, later proposes synonymy between *N. bruneri* and *N. flavida*, given the plasticity of the characters with which was diagnosed both species (2).

Some authors (3) propose as not valid the synonymy proposed earlier (2), based on morphological differences in the external genitalia of the male and include both species in the *Triatoma* genus, already considered as synonymous with *Nesotriatoma* in (2–4). The separation of both species and their inclusion are accepted in the *Triatoma* genus (5).

Posteriorly, the separation of the two species is maintained but based on phylogenetic analyzes include them in the *Nesotriatoma* genus (6). In a checklist of the current valid species of the subfamily Triatominae also consider valid *Nesotriatoma* genus (7). This genre is composed by the species *N. flavida*, *N. bruneri* and *N. obscura* (*flavida* complex). However, recently (8) by genetic analyzes published an article questioning the specific status of *N. bruneri* because this specie presented the same cytogenetic characteristics of *N. flavida* and an extremely low genetic distance (0.004) (8).

The specimens with proposed the separation of *N. flavida* and *N. bruneri* based on the morphological characters of the external genitalia of the males insects were sent by Dr W Torrealba (3). “Dr Torrealba sent us 5 specimens from the corresponding group. One specimen was identified as a typical *T. flavida* female captured in a human dwelling, and 4 specimens (two males and two females) captured in an animal burrow (?) of Pinar del Río Province, Guanahacabiles in the west of Cuba on 4-11-1979”.

With individuals from the same locality and captured on the same date a colony was established at the Universidad de La Habana, Cuba and with individuals of this colony was founded the colony of *N. flavida* at our laboratory in Mérida, Venezuela whose members were used in this work for analyses of external morphological characteristics and of the male external genitalia, with the aim of verifying whether the triatomines that form the colony were *N. flavida* or *N. bruneri*, in view of the polemic in the taxonomic status of these species.

## Materials and Methods

### Entomologic material

Nine males and nine females of *N. flavida* from the Laboratory of Entomology “Herman Lent” (HLEL), Venezuela, were examined, being maintained at 28 °C, 50% relative humidity, and fed hen blood. This colony was founded on 18-6-1980 with eggs, 3 females, 2 males, and 5 fifth instar nymphs brought by Dr Scorza from the colony identified as *Triatoma flavida* in the Universidad de La Habana, Cuba.

### Morphological analyses

The analysis of the external morphology of the adult body was carried out following the taxonomic keys (4), and the descriptions of both species (1, 5). The analysis of the morphology of the external male genitalia was done following the terminology employed and descriptions of the external male genitalia of both species (3–5).

Both morphological studies were observed with a Leica M205A stereoscopic microscope, the images were taken with a CMOS camera (EOS Rebel T3, Canon). The genitalia were dissected at the level of the two final abdominal segments, and processed in the following fashion: KOH (Eka, Chemicals) at 10% in a mortar and heated without boiling until soft, later treated according to treatment 1: 36h in phenol (90%, Honeywell Riedel-de-Haën) and 48h in guaiacol (Scharlau Chemicals, S.A.), or treatment 2: 72h both in the phenol and in the guaiacol. Finally, they were mounted in DPX (Industrias Químicas ERBA, C.A.) on a microscope slide and observed under a stereo microscope (Leica M205). The pictures were taken with a CMOS camera (Leica EC3) coupled to this microscope. The drawings of the borders both of the silhouette of the phallosome as well as its support in the male external genitalia were done using a raster graphics editor (Photoshop CS6, 13.0 ×64).

## Results

### External morphology of the body

**Figs. 1–7.**
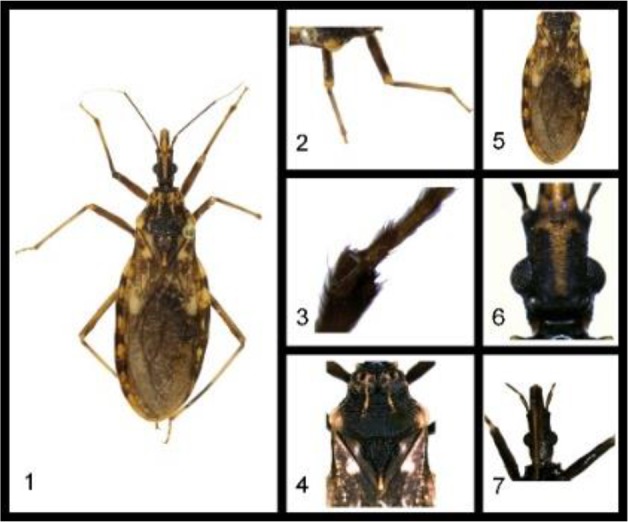
External morphology of the body of *Nesotriatoma. flavida*. 1: Dorsal view of complete male body. 2: Femur and tibia of the female. 3: Spongy groove on the distal end of the foreleg tibia. 4: Dorsal view of the pronotum and scutellum of the female. 5: Dorsal view of the scutellum and hemelytrons of the male. 6: Dorsal view of the male head. 7: Ventral view of male head

General color: clear brown. Body and corium are practically hairless, hairs scarce and very short. Femur: variable: from completely dark or completely light, or the proximal half dark and the distal light, with the presence or absence of denticles on the front legs. Tibias and distal parts of the end of the femur have a light color (Figs. 1, 2). Spongy grooves under-developed, present on the front legs of both males and females (Fig. 3). Pronotum is dark, except for the humerus, anterolateral angles, and the submedian carinae, which are of a yellow color. Pronotum has prominent anterolateral and humeral angles, anterior lobes with very noticeable lateral and discal tubercles and with yellow-edged halos, a butterfly figure at the union of the two lobes. On the front edge of the scutellum disk, 1+1 prominent tubercles pointed forwards and touching the back edge of the pronotum. Median region of the scutellum has noticeable transverse grooves (Fig. 4). Hemelytron is covering laterally part of the tergites and reaching the seventh urotergite, extensively spotted towards the back part and clearly defined dark stains on the front portion (Fig. 5). Proportion eye width: interocular distance 1:2 (Fig. 6). Third rostrum segment is shorter than the others. First antenna segment does not overshoot the peak of the clypeus (Fig. 7).

### Morphology of the male external genitalia

**Figs. 8–16.**
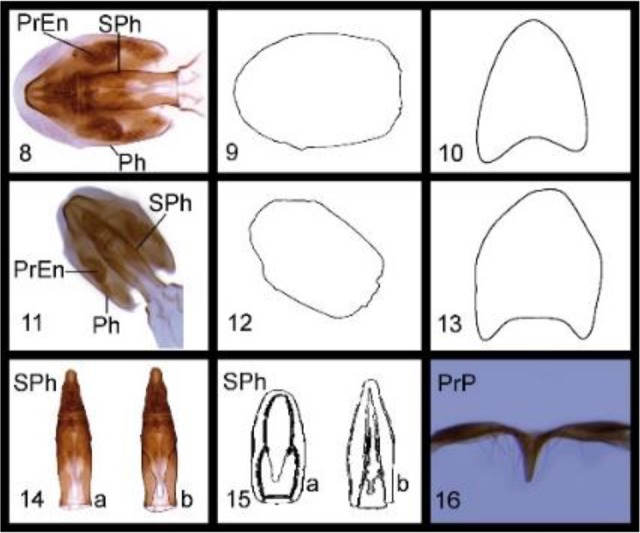
Morphology of external male genitalia. 8: Ventral view of phallosome, treatment 1 (see Materials and Methods). 9: Phallosome silhouette, treatment 1. 10: Phallosome silhouette of *N. bruneri* according to (3). 11: Ventral view of phallosome, treatment 2 (see Materials and Methods). 12: Phallosome silhouette, treatment 2. 13: Phallosome silhouette of *N. flavida* according to (3). 14: Phallosome support, a: treatment 1, b: treatment 2. 15: Phallosome support, a: *N*. *flavida*, b: *N. bruneri*, according to (3). 16: Median processes of pygophore. Ph: phallosome, PrEn: endosome process, SPh: phallosome support, PrP: median process of pygophore.

Phallosome lengthy, oval, and with concave base when the genitalia are maintained 36h in phenol and 48h in guaiacol (Figs. 8, 9, 10 (3)). Phallosome lengthy, hexagonal, and concave base when maintained 72h in both phenol and guaiacol (Figs. 11, 12, 13 (3)). The process of the endosome extends approximately 75% of its length from the base of the support of the phallosome, with a lengthy, very wrinkled and grooved appearance when the genitalia are maintained 36h in phenol and 48h in guaiacol (Fig. 8) as well as when maintained 72h in both the phenol and the guaiacol (Fig. 11). The phallosome support long, with cylindrical base, side edges converging towards the outer end of the base under either of the treatments of the genitalia (Figs. 14, 15 (3)). Median process of pygophore simple and pointed (Fig. 16).

## Discussion

The characters: length of the first antenna segment in relation to the clypeus, presence of spongy grooves, and ratio eye breadth to interocular distance as features that distinguish *N. flavida* from the new species he calls *N. bruneri* (1), all of which later (2) considers so variable that they do not justify the distinction between the species, and so he considers them synonymous. The external male genitalia of *N. flavida* was described as a quadrangular phallosome, phallosome support with parallel edges, semicircular vesica, and long endosome processes, while those of *N. bruneri* is described as being an oval phallosome, convergent edges of the phallosome support, ovoidal vesica, and short endosome processes (3). Examining the illustration was provided (Fig. 13), the phallosome of *N. flavida* was not quadrangular, but rather hexagonal (Figs. 11, 12). The endosome process extends from the middle part of the phallosome to the vesica in *N. flavida*, and indicated it in *N. bruneri*, but it is indistinguishable in such illustration (not showed). Although we observed that the phallosome support is pointed in both specimens: *N. flavida* described in our work (Figs. 14a,b) and in *N. bruneri* described, according to these authors the phallosome support is blunt in *N. flavida* and more pointed in *N. bruneri* (Fig. 15a,b) (3). Although we observed spongy grooves in female and males, the presence of spongy grooves varies according to whether the legs are frontal or medial and according to sex, the authors did not describe this structure in *N. bruneri*. They described the interocular distance as being double the eye width in both species (3). Finally, we observed that the first antenna segment does not reach the peak of the clypeus in any of the species (Fig. 1).

In our opinion the distinction between *N. flavida* and *N. bruneri* proposed (3) is poorly based, the description of the phallosome silhouette is erroneous, the imprecise illustration and distinction of the vesica as semicircular in *N. flavida* and ovoidal in *N. bruneri*, the poor illustration of the endosome process in *N. bruneri*, the imprecision with which the endosome process is considered “large”, and the absence in that paper of a comparative analysis between the species based on those characters that first distinguished the species and then later considered them synonymous given the phenotypical plasticity of the length of the first antenna segment with respect to the clypeus, the spongy grooves, and the relation between eye width and interocular distance (1, 2).

In the present work it was found that the specimens identified as *N. flavida* selected from the colony in our laboratory, formed from the same group of insects collected in Guanahacabiles in Pinar del Río Province in the west of Cuba (3), present the following features: 1) the first antenna segment does not overshoot the clypeus peak like that described in *N. flavida*, and in *N*. *flavida* and *N. bruneri* (1,3), 2) spongy grooves poorly developed, present in the forelegs of both males and females, as describe for *N*. *bruneri* (3), and differing from what observes as well developed grooves in the female and absent from the males of *N. flavida*, and missing in both sexes of *N. bruneri*, (1) and also differing from what observe in finding the grooves present on the front and middle legs of the males and variable presence on the forelegs of the *N*. *flavida* female (3), 3) ratio eye width–inter-ocular distance 1:2, similar to what describes for *N. bruneri*, for *N. flavida* and *N. bruneri*, and differing from the description in for *N. flavida* (1:3) (1,3), 4) when the genitalia were treated 36h in phenol and 48h in guaiacol, the phallosome silhouette is found to be oval, similar to that described for *N. bruneri* (3), (Fig. 10), while on the other hand, phallosome has a hexagonal silhouette when treated 72h in phenol and 72h in guayacol, similar to that described as “quadrangular” in *N. flavida* (Fig. 13), 5) phallosome support base cylindrical, pointed peak and convergent edges irrespective of the phenol and guaiacol treatments, similar to the cylindrical support base described for *N. flavida* (3), (Fig. 15a), and to the pointed peak and convergent edges of *N. brunei* by these same authors (Fig. 15b), and 6) endosome processes long and wrinkled in either of the different times in phenol and guaiacol, for *N. bruneri* (3) (Table 1). In the present work, differences were found in characteristics of the external male genitalia according to as to how they were treated in phenol and guaiacol, which exemplifies the importance of a detailed description of the treatment protocols of the genitalia, an aspect not treated (3).

**Table 1.** Comparison of external morphological characters of the body and of the external male genitalia of *Nesotriatoma flavida* and *Nesotriatoma bruneri*

**Character**	**(1)**		***N. flavida* (4)**	**(3)**		***N. flavida* Present work**
	
	***N. flavida***	***N. bruneri***		***N. flavida***	***N. bruneri***	
**First antenna segment size**	Sh	L	Sh	Sh	Sh	Sh
**Spongy groove distribution**	F:An	F:An	M:An, Md; F:V	M:An, Md; F:V	M:An, Md; F:An, Md	M:An; F:An
**Spongy groove degree of development**	D	N/D	N/D	N/D	SD	SD
**Ratio eye width: interocular distance**	1:3	1:2	1:3	1:2	1:2	1:2
**General body color**	Brown and ochre	Mottled brown	Light hazel	Light brown	Dark mottled hazel	Light hazel
**Form of distal protuberances of anterior pronotal lobe**	Sm, Rd, Prm	Prm	Rd, Subc	N/D	N/D	Prm
**Form of lateral protuberances of anterior pronotal lobe**	Sm, Rd, Prm	Prm	Rd, Subc	N/D	N/D	Prm
**Presence of denticles in anterior and median femurs**	A	P	P	P	P	V
**Form of phallosome**	N/D	N/D	N/D	L, Hx, BsCc	L, Ov, BsCc	tr1:L, Ov, BsCc tr2:L, Hx, BsCc
**Form of phallosome support**	N/D	N/D	N/D	L, BsCy, BrLPr	L, BsCy, BrLCv	tr1:L, BsCy, CvLE tr2:L, BsCy, CvLE
**Form of endosome processes**	N/D	N/D	N/D	L, Al	Sh, W	L, W
**Median processes of pygophore**	N/D	N/D	N/D	N/D	N/D	S, Pn

Sh: short, L: long, F: female, M: male, An: anterior, Md: median, V: variable, D: developed, N/D: not described, SD: slightly developed, Sm: small, Rd: round; Prm: prominent, Subc: subconic, A: absent, P: present; Hx: hexagonal, BsCc: concave base, Ov: oval, BsCy: cylindrical base, PrLE: parallel lateral edges, CvLE: convergent lateral edges, Al: aliform, W: wrinkled, S: simple, Pn: pointed, Sth: smooth, tr1: treatment 1, tr2: treatment 2. Numbers in parenthesis correspond to citations in References.

Our proposal for the revalidation of the synonymy between *N. flavida* and *N*. *bruneri* is based on the following facts: 1) according to our results and the corresponding ones (1, 3), the morphological characteristics such as length of the first antenna segment, presence of spongy grooves, ratio eye width: interocular distance, general body color, and denticles of femur are quite variable, 2) in our results the form of the male external genitalia varies according to the hours of treatment in phenol and guayacol, showing characters like either what described for *N. flavida* or for *N. bruneri* (3).

Based on the sequence of the gene 16S rRNA, was found differences (ca. 1.37%) between *N. flavida* and *N. bruneri* (6). Although there is no agreement about the amount of difference at the sequence level that would constitute a proof of distinction between species, the differences at the sequence level of the 16S rRNA gene (not a protein coding gene but an RNA structural gene with a slow rate of change), would not alone permit sustaining the thesis of different species. This kind of evidence is valid if it lends support to other characters like morphological, ecological, behavioral, etc. Since there is no evidence of these differences, the distinction based solely on the 16S rRNA gene sequence (6) is for the moment insufficient to classify *N. flavida* and *N. bruneri* as different species.

The genetic variability of *N. flavida* and *N. bruneri* were analyzed by means of the RAPD-PCR technique and found differences between the species (9). This technique is not valid to separate species, but rather is used for polymorphism analysis or intraspecific variation, and so found does not invalidate the synonymy between these two species.

By means of an antenna phenotype discriminating analysis manage to separate *N. flavida* from *N. bruneri* (10), but since they do not find differences upon comparing the same sex between the two species, the separation should not be taken as definitive, but rather subject to later molecular, ecological, and morphological analyses to clarify the taxonomic status of both species. Given the ambiguity of the differences found with the discriminating analysis of the antenna phenotype reported by these authors, we also consider that their results do not constitute evidence that invalidates the synonymy between *N. flavida* and *N. bruneri*.

Based on the foregoing exposition, we consider that maintaining the synonymy between *N. flavida* and *N. bruneri* is more solidly justified than considering them different species. The synonymization of *N. bruneri* with *N. flavida* is of utmost importance to public health in Cuba because the country ceases to have four species of triatomine [*Bolbodera scabrosa*, *N. bruneri*, *N. flavida*, *T. rubrofasciata*] and has only three species now, being *N. flavida* with wide geographic distribution. Furthermore, the synonymization allows clarifying evolutionary questions, as for example the occupation of the Antilles by this species associated with a species of rodent about 14.8 to 18.8 Ma (7, 11).

## Conclusion

Based on the body external morphology and the male external genitalia of *N. flavida* it is concluded that *N. flavida* and *N. bruneri* are synonymous species.
